# Effects of Wet/Dry-Cycling and Plasma Treatments on the Properties of Flax Nonwovens Intended for Composite Reinforcing

**DOI:** 10.3390/ma9020093

**Published:** 2016-02-03

**Authors:** Heura Ventura, Josep Claramunt, Antonio Navarro, Miguel A. Rodriguez-Perez, Mònica Ardanuy

**Affiliations:** 1 Departament d’Enginyeria Tèxtil i Paperera, Universitat Politècnica de Catalunya-BarcelonaTech, Colom 11, Terrassa 08222, Spain; heura.ventura@upc.edu; 2 Departament d’Enginyeria Agroalimentària i Biotecnologia, Universitat Politècnica de Catalunya-BarcelonaTech, Av. del Canal Olímpic 15, Castelldefels 08860, Spain; josep.claramunt@upc.edu; 3 Departament d’Enginyeria Química, Universitat Politècnica de Catalunya-BarcelonaTech, Colom 1, Terrassa 08222, Spain; antonio.navarro@upc.edu; 4 Cellular Materials Laboratory, Condensed Matter Physics Department, Universidad de Valladolid, Paseo Belén 4, Facultad de Ciencias, Valladolid 47011, Spain; marrod@fmc.uva.es

**Keywords:** plasma treatment, cellulose fibers, surface functionalization

## Abstract

This research analyzes the effects of different treatments on flax nonwoven (NW) fabrics which are intended for composite reinforcement. The treatments applied were of two different kinds: a wet/dry cycling which helps to stabilize the cellulosic fibers against humidity changes and plasma treatments with air, argon and ethylene gases considering different conditions and combinations, which produce variation on the chemical surface composition of the NWs. The resulting changes in the chemical surface composition, wetting properties, thermal stability and mechanical properties were determined. Variations in surface morphology could be observed by scanning electron microscopy (SEM). The results of the X-ray photoelectron spectroscopy (XPS) showed significant changes to the surface chemistry for the samples treated with argon or air (with more content on polar groups on the surface) and ethylene plasma (with less content of polar groups). Although only slight differences were found in moisture regain and water retention values (WRV), significant changes were found on the contact angle values, thus revealing hydrophilicity for the air-treated and argon-treated samples and hydrophobicity for the ethylene-treated ones. Moreover, for some of the treatments the mechanical testing revealed an increase of the NW breaking force.

## 1. Introduction

During the last few years, there has been growing interest in the development of more sustainable and ecofriendly composite materials. In this sense, natural fibers have been proposed as an interesting option for the reinforcement of not only polymeric matrices [1,2,3,4] but also for other ceramic matrices such as cements [5]. Cellulosic or vegetable fibers are non-hazardous, renewable, biodegradable, and have low density and well-balanced stiffness, toughness and strength, allowing the development of composite materials with good performance at relatively low cost [5].

Despite all of the aforementioned advantages, one of the main drawbacks of these fibers is their high hydrophilicity, which makes them very sensitive to water. Cellulosic fibers have a cellulose structure and other hydrophilic components with an affinity for water, which favors penetration of the same in the amorphous regions of the fibers. The amount of moisture that can be absorbed, depending on environmental conditions, can vary between 5% and 20% by dry weight. Therefore, one of the main problems to be solved for composites reinforced with cellulosic fibers is the fiber-matrix interaction which is weakened by the dimensional changes of the fibers due to the absorption and desorption of water. One possible treatment used to minimize the dimensional changes due to this effect is the previous modification of fibers with a wet/dry treatment. It is well known that drying and rewetting cycles in water cause shrinkage of the cellulosic fibers and a reduction in water retention values due to the formation of hydrogen bonds in the cellulose [6,7].

On the other hand, this hydrophilicity also affects the interface adhesion with matrices. In general terms, the interaction with cementitious matrices is positive but becomes a problem for most polymeric matrices which are more hydrophobic. The treatments commonly used to improve the compatibility and surface contact and hence the fiber-matrix adhesions are chemical or enzymatic. Nevertheless, these techniques are costly and complex and use high amounts of water and chemicals, which make them poorly applicable to consumer goods. One method that requires less energy, reduces the use of hazardous substances and avoids the generation of wastes is plasma surface treatment, which confers various surface properties, such as increased reactivity of the surface. Plasma treatments physically and chemically modify the fiber surface without any variation in the intrinsic properties of the fibers. The main advantages of the use of plasma technologies are: the minimal use of chemical products (gases or precursors) and energy (no need to dry); water is not required, thus avoiding residual waters and their treatment; and they can be applied in continuous processes. The main limitations are due to difficulties in controlling the atmospheric plasma and the investment costs, despite the latter being progressively reduced with the increasing demand. On the other hand, plasma-induced grafting and plasma polymerization techniques can provide diverse surface properties [8,9]. It is possible to achieve increased hydrophilicity in cellulosic fibers [10,11,12,13] or to obtain hydrophobic cotton fibers with plasma polymerization processes [14,15].

The aim of this study is to evaluate the effects of more eco-friendly methods for the functionalization of flax nonwovens (NWs) intended for composite reinforcing. On the one hand, plasma treatments can be considered as eco-friendly solutions due to the minimal use of chemicals, lower use of energy and no use of water, as stated before. Moreover, these plasma treatments can be appropriately optimized to affect only the fiber surface, thus avoiding the major loss of mechanical properties (unlike other more aggressive chemical treatments). On the other hand, the wet/dry cycling has the advantage of being a very simple treatment that only requires water (with no other hazardous substances) to achieve better fiber stabilization. As aforementioned, the effects of these treatments on cellulosic fibers have already been studied in the literature. However, to the authors’ knowledge, no studies dealing with their combination have already been done. Both the stability and the adhesion of the reinforcement are important factors that can affect to the durability of a composite. Therefore, in this study, various surface plasma treatments with previous wet/dry cycling treatments have been applied and evaluated by means of morphology and chemical characterization, water absorption behavior, and thermal and mechanical properties.

## 2. Results and Discussion

The original NW (SC) was treated with several methods: wet/dry cycling (C), low pressure argon plasma (Ar), low pressure ethylene plasma (Et) and air corona plasma (Cr), in several conditions. Table 1 shows the references of the samples and a description of the treatments and combinations applied.

**Table 1:** Sample references and description of the treatment conditions applied.

Reference	Wet/Dry Treatment (Cycles)	Corona-Plasma (min)	Argon Plasma (min)	Ethylene Plasma (min)
NW SC	0	-	-	-
NW C	4	-	-	-
NW C-Et10	4	-	-	10
NW C-Ar5	4	-	2.5 + 2.5	-
NW C-Ar10	4	-	5 + 5	-
NW C-Ar20	4	-	10 + 10	-
NW C-Ar30	4	-	15 + 15	-
NW C-Ar5-Et5	4	-	5	5
NW C-Ar5-Et10	4	-	5	10
NW C-Cr20	4	20	-	-
NW C-Cr1010	4	10 + 10	-	-
NW C-Cr1010-Et10	4	10 + 10	-	10

Note: Plasma treatments were applied to one side (*n*) or to both sides (*n* + *n*) of the NW, so *n* refers to treatment time per side.

### 2.1. Surface Characterization

#### 2.1.1. SEM Analysis

**Figure 1:**
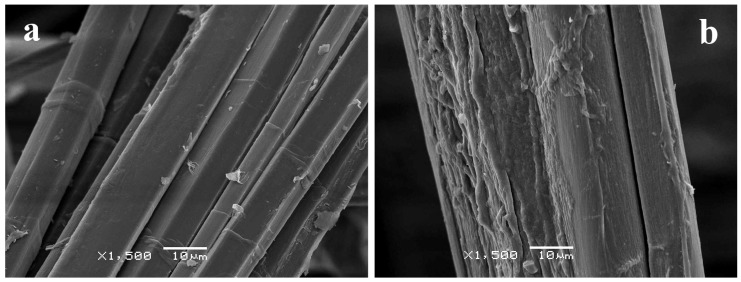
SEM images of (**a**) untreated; and (**b**) wet/dry cycled flax bundles taken at 10 kV and ×1500 magnification.

Surface morphology of the fibers was observed by Scanning Electron Microscopy (SEM). As shown in Figure 1, after the wet/dry cycling, the fibers presented a rougher surface while the untreated sample had the typical appearance of flax fibers with smoother surfaces. However, despite some shrinkage was expected, it could not be observed due to the high variability in the diameters of fibers.

**Figure 2:**
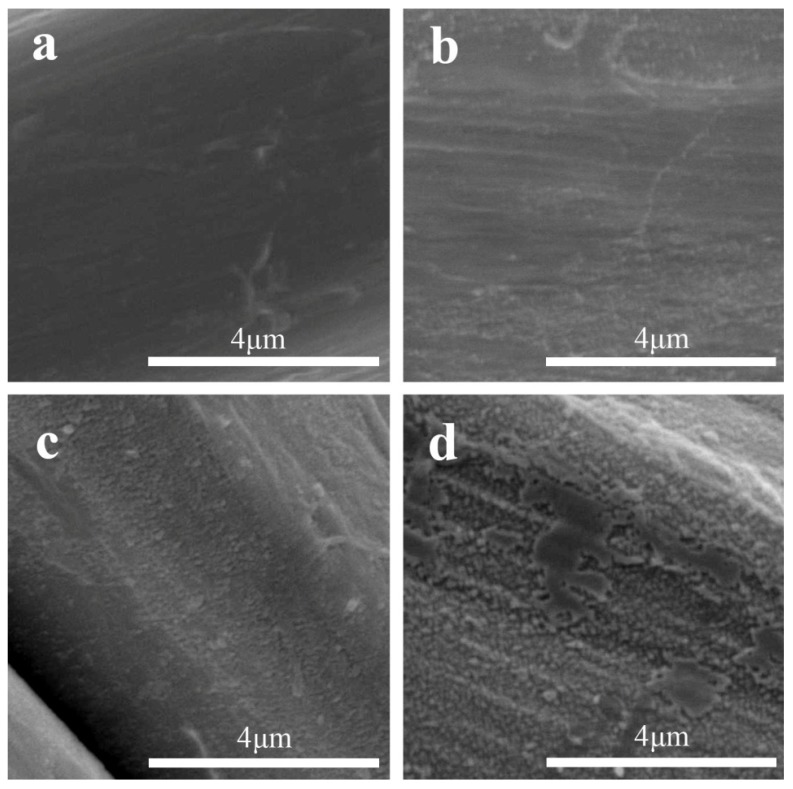
Effect on the surface roughness of the fiber due to increasing time in Ar-plasma treatment of samples: (**a**) NW C-Ar5; (**b**) NW C-Ar10; (**c**) NW C-Ar20; and (**d**) NW C-Ar30. SEM images were taken at 10 kV and ×10,000 magnification.

**Figure 3:**
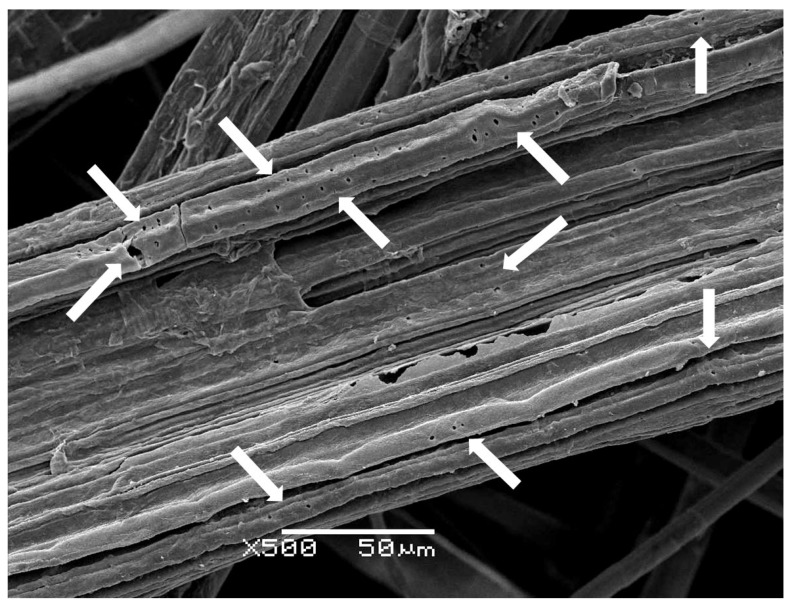
SEM image of the sample NW C-Ar30 taken at 10 kV and ×500 magnification. Arrows mark the craters formed.

The low pressure Ar treatment led to an increase in surface roughness when increasing the treatment time, as expected. Figure 2 shows these differences on the surface roughness of samples after 5, 10, 20 and 30 min of Ar-plasma treatment at high magnification. This is consistent with the fiber surface etching produced by Ar-plasma [16,17]. Moreover, some small craters of around 0.5–1 µm could be observed in the NW C-Ar30 sample (Figure 3), thus attributed to the aforementioned etching following longer exposure times.

**Figure 4:**
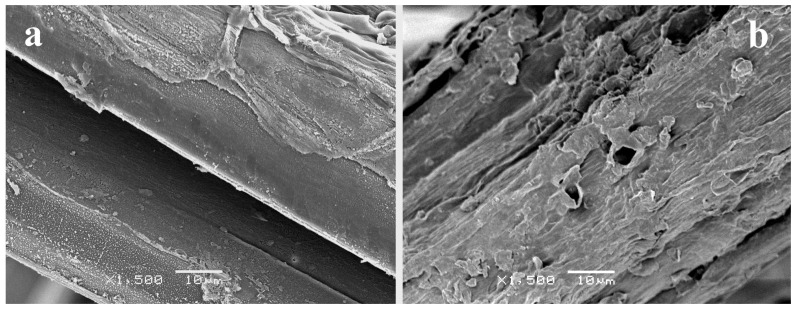
SEM image of surfaces of samples (**a**) NW C-Cr1010; and (**b**) NW C-Cr20 taken at 10 kV and ×1500 magnification.

Samples in which air corona plasma treatment was applied also presented high roughness (Figure 4), although some differences could be observed between treating the NW for 20 min on one side only (NW C-Cr20) and treating it for 10 min on each side (NW C-Cr1010). The NW C-Cr20 presented higher levels of damage on the fiber surface of the treated side, which was attributed to the higher etching caused by the plasma.

**Figure 5:**
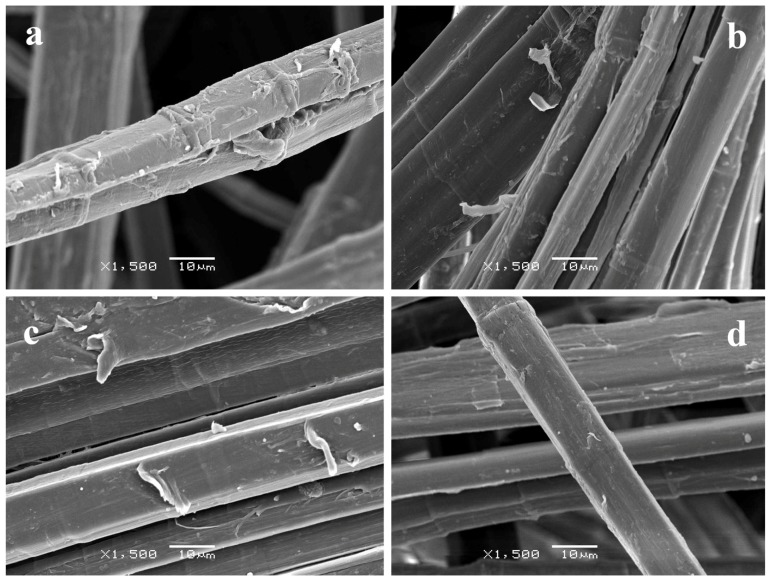
SEM image of surfaces of samples (**a**) NW C-Ar5-Et5; (**b**) NW C-Ar5-Et10; (**c**) NW C-Cr1010-Et10; and (**d**) NW C-Et10 taken at 10 kV and ×1500 magnification.

**Figure 6:**
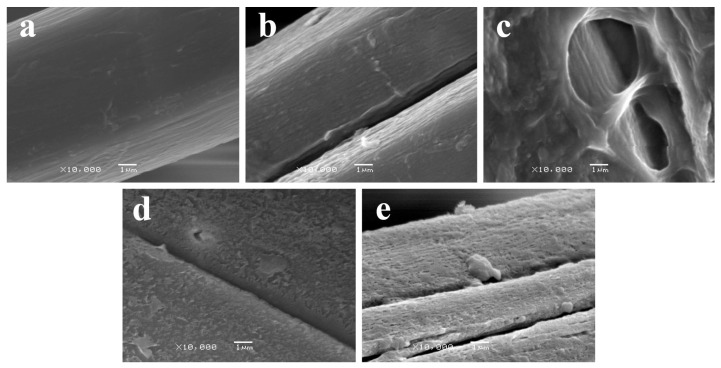
Comparative SEM images taken at 10 kV and ×10,000 magnification, of the surfaces of samples (**a**) NW C-Ar5; (**b**) NW C-Ar5-Et5; and (**c**) NW C-Ar5-Et10; and (**d**) NW C-Cr1010; and (**e**) NW C-Cr1010-Et10.

When using further low pressure Et-plasma treatments, fiber morphology did not change significantly. Only flake-like attachments could be observed, which were attributed to partial lifting of thin ethylene layers that polymerized on the surface of the fibers (Figure 5). The polymer deposition in the surface was more clearly observed in samples with higher roughness (see Figure 6).

#### 2.1.2. XPS Analysis

Plasma treatments generate new reactive groups on the surface and, at the same time, remove other chemical groups due to surface etching. Therefore, X-ray photoelectron spectroscopy (XPS) analyses are required to provide clear information about chemical composition. In the survey scans of XPS spectra, the major elements identified were carbon (C1s) followed by oxygen (O1s) with a negligible amount of nitrogen, as expected [17]. Elemental compositions and oxygen to carbon (O/C) ratios are presented in Table 2.

**Table 2:** Elemental composition percentage and oxygen to carbon (O/C) ratios for all the nonwoven (NW) samples obtained by X-ray photoelectron spectroscopy (XPS).

Reference	Elemental Composition (Atomic %)	O/C Ratio
	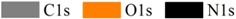	
NW SC	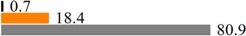	0.23
NW C	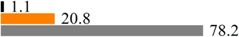	0.27
NW C-Et10	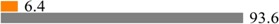	0.07
NW C-Ar5	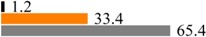	0.51
NW C-Ar10	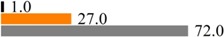	0.37
NW C-Ar20	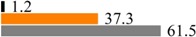	0.61
NW C-Ar30	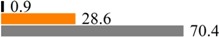	0.41
NW C-Ar5-Et5	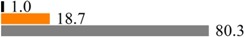	0.23
NW C-Ar5-Et10	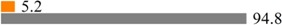	0.05
NW C-Cr20	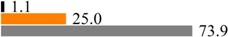	0.34
NW C-Cr1010	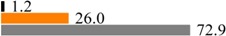	0.36
NW C-Cr1010-Et10	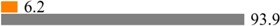	0.07

As far as the O/C ratio is concerned, it has to be pointed out that initial ratio of 0.23 was slightly increased to 0.27 due to the wet/dry cycling treatment. This could be attributed to the loss of lignin during the treatment process, since O/C ratios have been previously related to the amount of lignin in the literature [18].

Further treatment with low pressure Ar-plasma and air corona plasma increased O/C ratios, in agreement with previous work [19]. In general terms, plasma treatments produced an enhancement of the surface reactivity due to free-radicals, and/or energetic and reactive states of surface atoms. As a result, more oxygen atoms could be attached to the surface in post-plasma oxidation processes. In this case, Ar-treatments showed higher effectiveness of surface oxidation in the samples of the study. However, some inconsistencies were found in the Ar-plasma samples. With increasing treatment time, the O/C ratios were expected to increase, but samples NW C-Ar10 and NW C-Ar30 did not follow the expected trend. This could be associated with the aging of the treatment [9,10] produced due to unavoidable contact with the ambient air during storage and a longer time delay between preparation and the XPS analysis. For that reason, samples NW C-Ar10 and NW C-Ar30 were not considered for further analysis of XPS results. For air corona plasma-treated samples, NW C-Cr20 and NW C-Cr1010 had similar results; therefore, only NW C-Cr1010 was considered for further XPS analysis.

When further treatment with low pressure Et-plasma was performed, the C and H elements of the ethylene gas were expected to cover the surface, producing an increase of C1s, a reduction of O1s (note the drastic reduction of the O/C ratios), and even the fading of N1s for all samples treated with a final 10 min Et-plasma. This indicates that the plasma polymerization would have achieved good coverage of the fibers.

**Figure 7:**
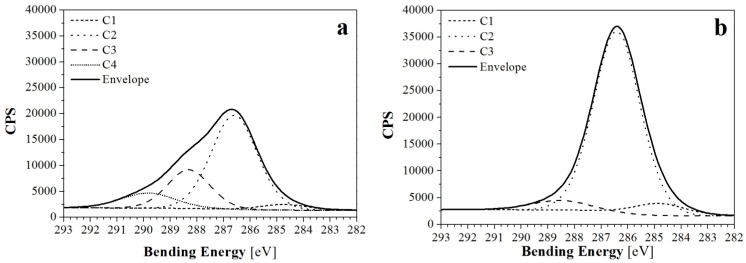
Deconvoluted curves of XPS C1s peaks of (**a**) NW C-Cr1010; and (**b**) NW C-Cr1010-Et10 samples. CPS stands for counts per second as a measure of the intensity.

**Figure 8:**
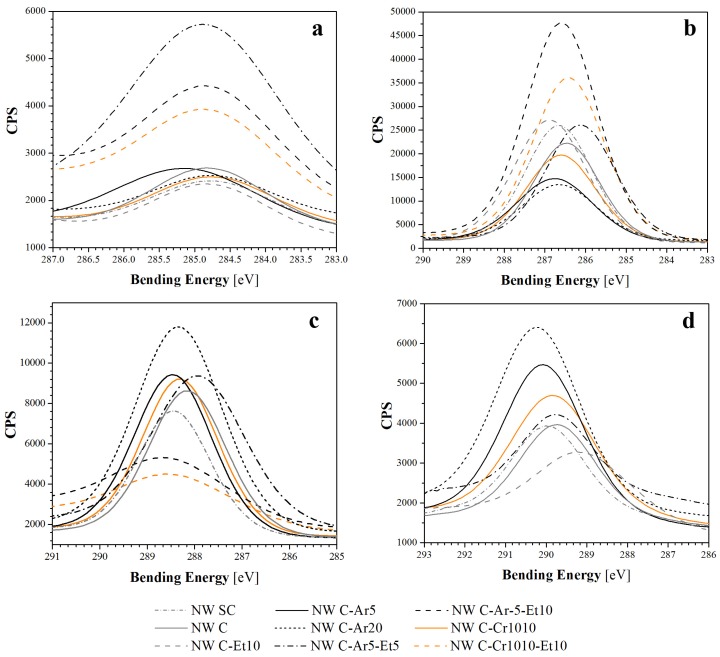
Comparative of the C1s peaks found in the deconvolution curves: (**a**) for the C1-peaks of all samples; (**b**) for the C2-peaks; (**c**) for the C3-peaks; (**d**) for the C4-peaks.

The analysis of high resolution spectrum for C1s revealed two kinds of composition (Figure 7) of the three main peaks (for the samples treated with 10 min of Et-plasma) or four main peaks (for the rest of samples). Those peaks were found at characteristic bending energies, corresponding with the results in the literature [17,18,19]: C1, which is mainly associated with C-C and C-H bonds, was found at 284.9 ± 0.2 eV; C2, associated with C-OH and C-O-C bonds, at 286.5 ± 0.4 eV; C3, associated with C=O and O-C-O bonds, at 288.4 ± 0.3 eV; and finally, C4, associated with O-C=O bonds, at 289.7 ± 0.5 eV. For comparative purposes, C1, C2, C3 and C4 peaks of all samples are shown in Figure 8. Differences in the height of these peaks revealed information about the loss or gain of specific bonds, and was thus compared with the reaction mechanisms to plasma treatments suggested in the literature [9,20].

No significant differences between NW SC and NW C samples were found. Comparison of C4 and C1 peaks (Figure 8a,d, respectively) showed equally low contents of O-C=O bonds and very similar contents for C-C linkages, respectively. Only a slight difference in C2 (Figure 8b) and C3 (Figure 8c) was found, showing lower C-OH and higher C=O and O-C-O contents for the treated sample, which could correspond to higher oxidation.

For low pressure Ar-plasma and air corona plasma treatments, similar trends were found, despite Ar-plasma treatments being more effective and samples revealing higher oxidation. On the one hand, Ar-plasma treatment of cellulosic fibers has been suggested to produce the cleavage of C-C bonds, which generates active groups capable of reacting with oxygen in post-plasma oxidation, thus leading to the formation of C=O and O-C-O groups [20], but also to a loss of C-OH groups. This would explain the lower C-OH curves in Figure 8b and the higher C=O and O-C-O curves in Figure 8c for the Ar-plasma-treated samples. On the other hand, the modification produced by the air corona plasma could be associated with the oxygen plasma reaction, since oxygen is the second major component in air. In this case, oxygen plasma has been associated with more intense C-O-C scission reactions and some cross-linking, thus producing a decrease in the C-OH and C-O-C peaks and an increase of O-C-O bonds [20,21]. Therefore, the results obtained for C=O and O-C-O groups in Figure 8c would confirm the post-plasma oxidation for Ar-treated samples and possible small cross-linking in the air corona plasma-treated samples.

Moreover, all samples presented similar contents of C-C and C-H bonds (Figure 8a), with the exception of samples NW C-Ar5-Et5, NW C-Ar5-Et10 and NW C-Cr1010-Et10, which showed higher contents of C-C and C-H bonds, probably due to the effect of ethylene plasma polymerization. In this sense, the ethylene layer showed the expected good adhesion, and also revealed minor oxidation of the samples. However, for the NW C-Et10 sample, the adhesion of this ethylene layer is expected to be worse due to the lack of a previous argon or corona activation step. This could explain the differences in the results for this sample with respect to the other Et-plasma-treated samples.

Finally, the O-C=O bonds (Figure 8d) should also be attributed to oxidation products, and, therefore, samples with oxidation presented high curves, while those with the ethylene layer did not show any signals.

**Figure 9:**
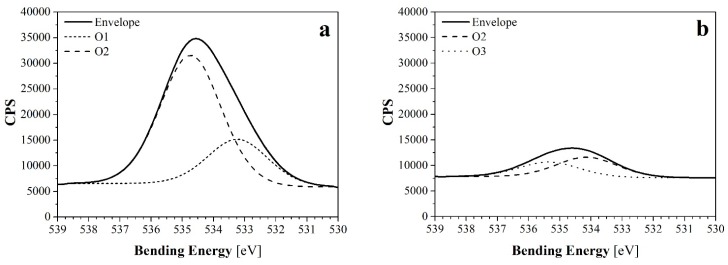
Deconvoluted curves of XPS O1s peaks of (**a**) NW C-Ar5; and (**b**) NW C-Ar5-Et10 samples.

The analysis of the O1s spectrum revealed differences between those samples with large exposure to Et-plasma and the rest of the samples. On the one hand, all samples treated for 10 min with Et-plasma presented a deconvolution with to peaks of lower intensity than the rest of the samples, at 534.0 ± 0.3 eV (O2) and 535.3 ± 0.2 eV (O3). Thus, they were associated to -O-C bonds and to absorbed water, respectively. As an example, the deconvoluted curves for the sample NW C-Ar5-Et10 have been represented in Figure 9b. On the other hand, the rest of the samples presented also a deconvolution of two peaks: one of lower intensity found at 533.1 ± 0.2 eV (O1), and other of higher intensity found at 534.6 ± 0.2 eV (O2). In this case, the peaks were associated to -C=O and -O-C, respectively. As an example, the deconvoluted curves for the sample NW C-Ar5 have been represented in Figure 9a.

The presence of -O-C bonds has to be related both to cellulose’s own nature and to the *in situ* and post-plasma oxidation phenomena. On the one hand, for the fibers treated with Et-plasma, the fact that its intensity is clearly lower (see the O2 peaks in Figure 9) is consistent with the C1 results, since the ethylene plasma polymerization would be hiding the fiber and avoiding the oxidation. The water presence related to O3 in these samples can be due to moisture absorption. On the other hand, for the rest of the samples, the intensities for the O2 peaks showed an increasing trend: first the untreated and the wet/dry cycled samples, then the corona plasma-treated samples, and finally the samples treated with Ar-plasma, which presented the higher intensities. A similar trend was observed in the O1 peak. In general terms, the larger intensities for those peaks can be related to the surface activation produce by the plasma treatments, what helped the *in situ* or post-plasma oxidation to fix the oxygen atoms to the fibers’ surface. The Ar-plasma treatment showed to be more effective than the corona plasma-treatment. In addition, the larger presence of -C=O groups in Ar-plasma treated samples (when compared to the original NW) is consistent with the results obtained for the C1s analysis.

### 2.2. Wetting Properties

#### 2.2.1. Water Retention Values

As shown in Table 3 and, as expected, the water retention value (WRV) decreased significantly after performing the wet/dry cycling treatment on the fabrics. Although high standard deviations of the values denote a certain irregularity that can be attributed to both the fibers and the treatments, the WRV were clearly reduced from 33.0% ± 1.7% for the untreated sample, and to half of this (average of 15.1% ± 6.4% of water retention) in all samples treated with the wet/dry cycling. This irreversible loss of water retention capacity and swelling, as a result of the wet/dry cycling, is related to the collapse and hardening of the outer walls of fibers [22].

Further plasma treatments did not show significant effects, since the variations presented were smaller than the deviations.

**Table 3:** Characterization results for NW.

Reference	WRV (%)	Moisture Regain (%)	Contact Angle (°)	Absorption Time	Breaking Force (N)	Strain at Break (%)
NW SC	33.0 ± 1.7	5.3	115	>1 h	12.0 ± 0.4	4.7 ± 2.2
NW C	14.8 ± 7.3	4.4	116	>1 h	11.4 ± 5.0	39.8 ± 13.0
NW C-Et10	10.8 ± 4.2	3.9	116	>1 h	12.7 ± 3.9	47.6 ± 0.8
NW C-Ar5	16.0 ± 5.8	4.0	108	30 min	14.9 ± 6.9	52.6 ± 11.5
NW C-Ar10	16.5 ± 9.0	4.2	94	15 min	15.8 ± 0.6	58.7 ± 2.0
NW C-Ar20	15.9 ± 6.4	4.2	94	36 s	16.7 ± 0.2	47.4 ± 9.8
NW C-Ar30	18.9 ± 5.3	4.0	83	4 s	13.5 ± 3.7	81.0 ± 11.8
NW C-Ar5-Et5	14.1 ± 7.5	4.1	117	>1 h	17.8 ± 5.7	47.9 ± 11.2
NW C-Ar5-Et10	12.2 ± 6.9	4.1	116	>1 h	29.0 ± 1.6	53.2 ± 0.8
NW C-CR1010	17.2 ± 5.9	4.2	80	50 ms	12.8 ± 2.0	41.7 ± 4.0
NW C-CR20	15.0 ± 8.1	4.1	74	5 ms	7.9 ± 1.1	56.9 ± 15.7
NW C-CR1010-Et10	15.0 ± 7.5	4.1	127	>1 h	28.7 ± 1.2	38.2 ± 2.4

#### 2.2.2. Moisture Regain

The moisture regain values of the samples ranged from 5.3% to 3.9%, with standard deviations of around 0.2. The highest value (5.3%) was found for the untreated NW (NW SC). After the wet/dry cycling treatment, there was a reduction in moisture regain, with a value of 4.4% for the NW C sample. This decrease is consistent with previous research, since the fiber is compacted, therefore presenting greater difficulty in absorbing any moisture present in the air.

Further plasma treatments helped to slightly reduce these values, which were similar in all cases, to around 4.1% moisture regain. No clear differences between the moisture regain of Ar-plasma and corona plasma samples were observed, or for further treatment with Et-plasma. Only in the case of the lowest value could a clear difference between the moisture regain of the previous step (4.4% for the NW C sample) and the further treatment with Et-plasma (3.9% for NW C-Et10) be observed, thus indicating that ethylene plasma polymerization helped to reduce the water uptake of the fibers, as expected for the creation of a more hydrophobic surface.

#### 2.2.3. Contact Angles

The initial contact angle of the untreated NW (NW SC) was around 115°, with completely hydrophobic behavior, since the drop remained stable on the surface for more than 1 h. The wet/dry cycled sample (NW C) did not show large variation in the contact angle nor absorption time, and a further treatment with Et-plasma polymerization for 10 min (NW C-Et10) failed to produce any change in these parameters.

However, when the Ar-plasma was used after the wet/dry cycling, the contact angles and the time taken to absorb the deposited drop were increasingly reduced when the treatment was longer, which resulted in general hydrophilicity. For Ar-plasma treatments between 5 and 30 min, contact angles were progressively reduced up to 28% and the time required for absorption of the deposited drop was also reduced from more than 1 h (previous conditions to the Ar-plasma, corresponding to the NW C sample) by up to 4 s. Therefore, higher hydrophilicity was obtained, which is consistent with the results in the literature [10,12,13,19]. An increase in water affinity is produced due to oxidation of the surface. After the creation of reactive groups on the fiber surface, these groups tend to react with air to form peroxide and other oxygen-containing groups [11,17,19,23,24,25,26]. The contact with air was unavoidable during manipulation after the plasma activation, and it was therefore expected that the adhesion of polar groups took place.

Nonetheless, for NW C-Ar5-Et5 and NW C-Ar5-Et10 samples, after a wet/dry cycling and 5 min treatment with Ar-plasma, the ethylene plasma polymerization took place on the surface of the NW fibers for 5 or 10 min. This led to the generation of a hydrophobic surface, which is consistent with the results in the literature [14], outbalancing the hydrophilicity generated with the previous step and recovering the initial contact angles (~116°) and absorption time (>1 h).

Similar results were observed for samples in which corona plasma was used, since oxidation species in the air used as plasma gas helped to form hydrophilic groups on the surface. Initial treatment with air corona plasma clearly increased the samples’ water affinity, reducing both contact angles and the time required to absorb the water droplet compared to the results of the only wet/dry cycled samples (NW C). However, the further treatment of Et-plasma polymerization helped to seal the fibers’ surfaces against water; as a result, both the contact angle and absorption time increased, corresponding to a more hydrophobic surface.

### 2.3. Thermal Stability

**Figure 10:**
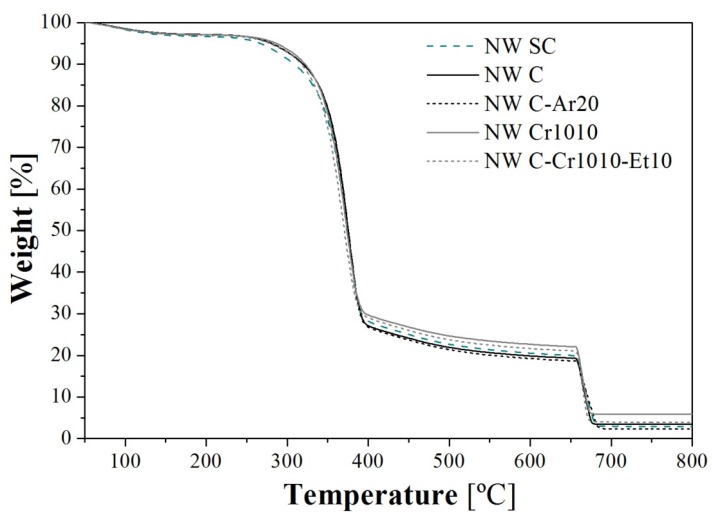
TGA curves of selected samples. Curve NW C-Ar20 can be considered the representative curve for all Ar-plasma-treated samples (NW C-Ar5, NW C-Ar10, NW C-Ar30, NW C-Ar5-Et5, and NW C-Ar5-Et10) due to their similarity.

Thermogravimetric analysis (TGA) was performed in all samples, obtaining similar curves in all cases (see selected examples in Figure 10); the main results are summarized in Table 4.

**Table 4:** Thermogravimetric analysis (TGA) results for the NW.

Reference	Moisture Loss (%)	Inflection Point (°C) 2nd Step	Inflection Point (°C) 3rd Step	Solid Residue (%)
NW SC	2.9	375	677	2.9
NW C	2.5	376	677	3.5
NW C-Et10	2.6	376	675	2.3
NW C-Ar5	2.5	374	677	3.0
NW C-Ar10	2.5	375	676	2.6
NW C-Ar20	2.6	375	677	2.3
NW C-Ar30	2.5	376	674	2.5
NW C-Ar5-Et5	2.5	373	673	2.9
NW C-Ar5-Et10	2.5	372	673	2.9
NW C-CR1010	2.5	374	675	5.9
NW C-CR20	2.5	376	673	3.3
NW C-CR1010-Et10	2.5	369	672	3.9

Note: Moisture or water loss is attributed to the 1st inflection on the TGA curve up to 125 °C.

From these curves, a slight improvement in stability due to the fiber treatment was observed between 250 and 350 °C, with the original NW SC presenting higher weight loss (around 10% higher) compared with all of the other treated samples. Between 375 and 650 °C, however, only small differences were observed, which were mainly attributed to the degradation of lignin [27]. On the other hand, between 675 and 750 °C, some differences were recorded, corresponding with the final residue. When comparing the untreated sample (NW SC) with the wet/dry cycled sample (NW C), an increase of the solid residue by 18% was recorded. Further treatments with Ar-plasma and Ar-plasma and Et-plasma did not present better results than the NW C sample. In fact, increasing the treatment time of Ar-plasma progressively reduced the solid residue, thus resulting in worse thermal stability, with results that were no better than those for the Et-plasma treatments. Nevertheless, corona treatment seemed to stabilize the fibers, since the solid residue was better than the original value in all cases. The best result was obtained for the NW C-Cr1010 sample, in which the solid residue was almost double that of the NW SC sample.

Despite the fact that only a small variation in thermal stability could be observed in this study, it is possible to find evidence of thermal stability improvement of natural cellulosic fibers by means of plasma treatment in the literature. Relvas *et al*. [27] found differences of around 14% in the residual weight after an equivalent TGA assay in quiscal fibers between 400 and 800 °C. The higher thermal stability was attributed to the formation of oxygen containing groups, which are more stable, thus avoiding complete degradation.

### 2.4. Fiber Crystallinity

**Figure 11:**
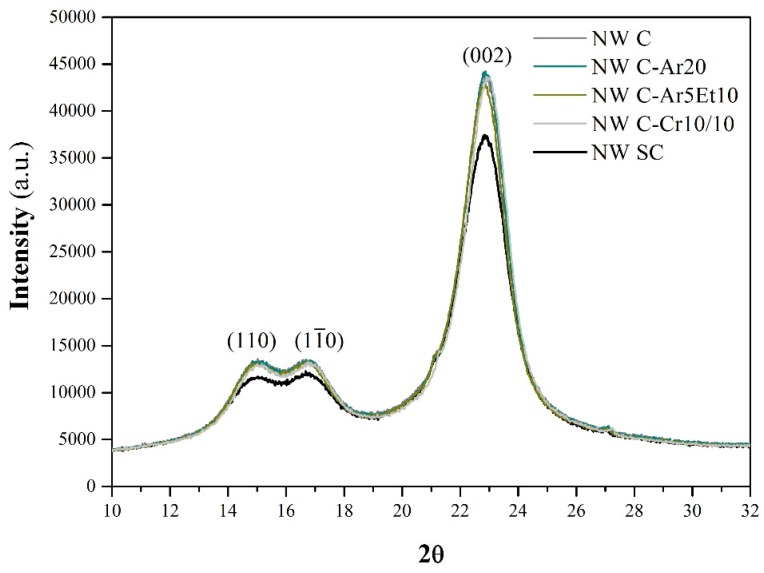
X-ray diffraction patterns of some selected treated and the untreated samples.

Plasma treatments are expected to modify only the fibers’ surface. The XPS analysis confirmed that this chemical modification was achieved. However, in order to check that the plasma treatments did only affected its surface chemistry, the crystalline structure was observed by means of X-ray diffraction (XRD). The results of the assays are shown in Figure 11.

On the one hand, a double-headed peak can be observed around 2θ = 16°, that corresponds to (110) and (1$\bar{1}$0) crystallographic planes. The appearance of this double-headed peak is related to high cellulose content, as expected for flax fibers [28]. On the other hand, a higher peak that corresponds to the (002) crystallographic plane of cellulose can also be observed at 2θ = 22.6°. The crystallinity indexes for the cellulose (CrI) have been calculated in order to compare the results. The untreated fibers (NW SC) presented a CrI of 81.0%. The rest of the samples, which have the wet/dry cycling treatment in common, presented very similar CrI, around 83.1% ± 0.4%, which is significantly higher.

The increase of the CrI after a wet/dry cycling treatment is associated with a higher packing on the cellulose crystals [29].

Furthermore, the XRD patters in Figure 11 also reveal that the different plasma treatments did not perform significant modifications on the fibers’ crystallinity, since it affected mainly the fibers’ surface. Despite some microstructural alterations of natural fibers due to plasma treatments having been reported in the literature (Morshed *et al.* [30] reported an increase on crystallinity and crystal size, for instance), this effect could not be observed in this study.

### 2.5. Mechanical Properties

Tensile tests were performed to the NWs in order to evaluate the reinforcing capacities of their structures. Therefore, due to the NW nature, the mechanical behavior is more similar to the deformation of a net than to the deformation of a solid, where the entanglement degree of the structure plays a key role. During the tensile test, the structure starts to elongate, the fibers are aligned and the entanglement points are stretched up to a maximum force. From this maximum point, the entanglement points are progressively broken, the strength progressively lost, and the aligned fibers start to progressively slide up to a total disentanglement of the section of the specimen. This means that the breakage is slow instead of abrupt; therefore, it is difficult to determine a breaking point. Due to that, this maximum has been considered as the breaking point. Breaking force and strain at break values, given in Table 3, can be considered as design parameters. High standard deviations reveal the complexity of characterization of NW due to their unavoidable heterogeneity that is added to the irregular nature of the bast flax fibers used in the study.

**Figure 12:**
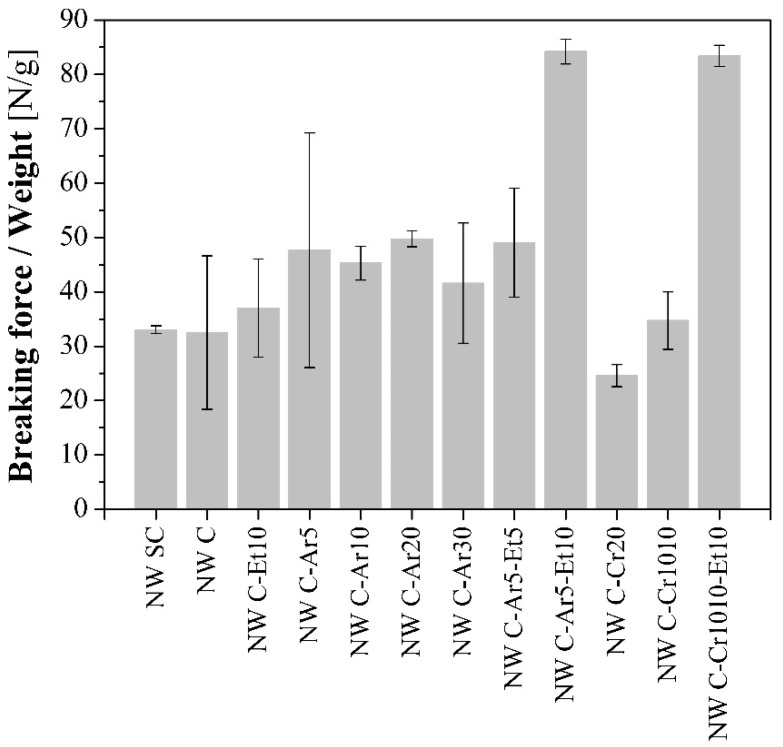
Breaking force of the NW normalized by the sample weight for comparative purposes only.

In order to understand the results and only for comparative reasons, the breaking force values were normalized by the weight of the specimens (Figure 12).

The wet/dry cycling did not reveal a clear effect in the NW resistance. However, the Ar-plasma seemed to increase the NW resistance when the treatment time was increased up to 20 min. The mechanical improvement of the NW structure after the plasma treatment can be attributed to higher inter-fiber friction. Plasma treatments enhance the roughness of the fibers’ surface due to the etching, thus leading to higher friction between fibers [31]. Therefore, it becomes more difficult to separate the entangled fibers, which leads to increased breaking force [32]. However, sample NW C-Ar30 presented a lower value, probably due to the stress concentration effect of the craters observed previously (Figure 3). Plasma corona-treated samples presented similar or lower resistance than the original NW. In this case, despite higher roughness is also achieved, the fiber damage generated during the process outweighs the enhancement of the inter-fiber friction. Thus leads to higher fiber breaking during the tensile test, and therefore, to lower breaking forces. In Figure 12, it can be observed that the results are clearly worse for the sample only treated in one side (NW C-Cr20), in which the treatment was clearly more aggressive (Figure 4). Furthermore, in the literature, Morshed *et al.* [30] pointed out that oxygen plasma caused higher stress than argon plasma, which weakens the fiber, affecting its strength and durability; this is consistent with the effects observed in the current study, since oxygen plasma is comparable to the air corona plasma treatment used here.

Further Et-plasma treatment caused plasma polymerization on the surface of the fibers. In samples with a highly activated surface (after the Ar-plasma or corona plasma treatments), the ethylene created a polymeric layer with good adhesion, thus improving the mechanical properties of the NW with similar results in samples NW C-Ar5-Et10 and NW C-Cr10 10-Et10. Thinner ethylene layers due to shorter exposure times, such as those obtained in NW C-Ar5-Et5, had less of a reinforcing effect. In the sample in which the surface was not activated (NW C-Et10), the reinforcing effect of the aforementioned layer was barely observed.

In what the strain is concerned with, a clear difference between the untreated samples (NW SC) and all the rest has been observed. The wet/dry cycling seems to be responsible of the higher flexibility and deformation of the NW structure. During the treatment, the cleaning of the fibers reduces the stiffness of the structure, thus helping to enhance the deformation capability of the net and, therefore, to achieve higher strain. Attending to the high irregularity of the fibers and the structure, no clear trends can be observed in to what effects the plasma treatments are concerned.

### 2.6. Considerations for Reinforcement Applications

Overall, on the one hand, it has been observed that wet/dry cycling is a good method to stabilize fibers against changes in humidity. Ar-treatment of up to 20 min is a good way to increase the hydrophilicity when considering NW for the reinforcement of cementitious matrices. Corona treatments would also be helpful in this sense but seem to produce greater damage to the fibers.

On the other hand, if conventional non-polar polymeric matrices (such as PP) are to be used, treatments of stabilization, activation and further plasma polymerization (such as those performed for NW C-Ar5-Et10) should be considered.

However, the requirements of the treatment will depend on the nature of the matrix to be used; some of the treatments presented here could be useful in the reinforcement of other more polar matrices.

## 3. Experimental Section

### 3.1. Materials

Flax fibers with an average length of 6 cm were provided by Fibres Reserche Development, of the Technopole de l’Aube en Champagne (Troyes, France). For plasma treatments, argon gas (99.999% of purity) and ethylene gas (99.99% of purity) were acquired from Air Liquide España (Madrid, Spain).

### 3.2. Nonwoven Preparation

NW fabric samples were prepared on a DILO OUG-II-6 pilot plant of double needle-punching machine, equipped with universal card clothing, a cross-lapper, a batt feeder and a needle-punching loom (DILO Group, Eberbach, Germany). The flax fibers were first opened and carded to form a thin web, which was laid by the cross-laying method to form batts. These batts were consolidated on the needle-punch to form the nonwoven mats. The optimized NW structure of 284 ± 23 g/m^2^ and thickness of 1.9 ± 0.1 mm with high entanglement was prepared. The processing parameters to prepare these nonwovens were evaluated in previous research [33].

### 3.3. Nonwoven Treatment

#### 3.3.1. Wet/Dry Cycling Treatment

The wet/dry cycling treatment (C) was performed by repetition of a two-step process. For the first step, NW were soaked in distilled water at 60 °C for 70 min under sonication. For the second step, the wet NW were dried in an oven with air recirculation at 105 °C for 16 h. A total of four wet/dry cycles applied to the samples.

#### 3.3.2. Plasma Treatments

Low pressure and corona plasma treatments were carried out in this study. Table 1 previously showed the reference and main characteristics of the treatments used.

For low pressure plasma, the NWs were treated by means of a Europlasma Junior Advanced PLC radiofrequency (RF) plasma reactor (Europlasma, Oudenaarde, Belgium), operating at 13.56 MHz and using argon (Ar) and ethylene (Et) gases at constant flow rate of 20 and 30 sccm, respectively. The plasma treatment procedure was as follows: first, the NW specimen was introduced into the reactor chamber and a vacuum of 50 mtorr was applied; then, the controlled flow of gas was injected, and the pressure inside the reactor was maintained at around 150 mtorr. At this point, the RF 50W-power plasma was initiated, and the NW specimen was exposed for the time previously specified in Table 1. Samples treated with both gases were first treated with argon, which was purged before further treatment with ethylene gas. This double treatment was performed without opening the reactor chamber (with no vacuum loss).

Corona plasma treatments (Cr) were carried out by means of an Ahlbrandt FG-2 corona plasma (Ahlbrandt System GmbH, Lauterbach, Germany) using air as a plasma gas. The distance between the electrode and the fabric was adjusted to 10 mm and power, speed and incident current were kept constant during the treatment at 400 W, 20 rpm and 2 A, respectively. Treatment time is also specified in Table 1.

### 3.4. Characterization of the NW Fibers

#### 3.4.1. Surface Characterization

Both the morphology and chemical composition of the fiber surface of the treated and untreated fibers was studied.

For the morphology analysis, images of the surface of the fibers were obtained by means of a JEOL-820 SEM (JEOL USA Inc., Peabody, MA, USA).

For the chemical composition, the XPS technique was used. XPS measurements were performed in a SPECS system (SPECS GmbH, Berlin, Germany), equipped with a monochromatic Al Kα anode XR50 operating at 100 W and a Phoibos 150 MCD-9 detector. The pass energy was set at 25 eV. The following sequence of spectra was recorded: survey spectrum taken at 1 eV, and high resolution spectra of C1s, O1s and N1s taken at 0.1 eV. The charge correction was performed with the C1s peak at 284.8 eV. A pressure lower than 10^−7^ Pa was maintained in the chamber. The relative error for XPS determination is about 0.5%. Each sample was analyzed at two different places and the average composition was calculated. Atomic fractions (%) were calculated using peak areas normalized on the basis of acquisition parameters after background subtraction. The O/C ratios were calculated considering the atomic fractions.

#### 3.4.2. Wetting Properties

For characterization of the wetting properties of all the NW samples, water-retention values, moisture-regain values and contact angle assays were used.

WRV were determined based on a standard ASTM D 2402-01 [34]. To ensure the water saturation of the samples, the specimens underwent vacuum-assisted immersion for 4 h. To remove excess water, the samples were centrifuged at 2600 rpm for 15 min. Moisture-regain values were determined following the DIN 54351 standard [35].

The static contact angle measurements were performed with a Krüss DSA 100 goniometer (KRÜSS GmbH, Hamburg, Germany). NW samples were laid flat on a clean, dry glass support without mechanical stress and 5 μL water droplets were deposited on the fabric surface. The time taken to absorb these water droplets was recorded. Contact angles were obtained from further analysis with the Drop Shape Analysis DSA3 software (KRÜSS GmbH, Hamburg, Germany).

#### 3.4.3. Thermal Stability

The thermal stability was determined by TGA using the Mettler TGA/SDTA 861 equipment (Mettler-Toledo S.A.E., L’Hospitalet de Llobregat, Spain). Samples weighing about 5.5 mg were heated in the temperature range from 25 to 750 °C with a heating rate of 20 °C/min under nitrogen atmosphere (at a flow of 60 mL/min).

#### 3.4.4. Fiber Crystallinity

X-ray diffractograms were obtained in a Bruker Discover D8 X-ray diffractometer (Bruker Company, Billerica, MA, USA), at a scan speed of 5.0 degree/min, in a scan range (2θ) from 5 to 50°, and with an X-ray tube producing monochromatic Cu Kα radiation.

The CrI, which corresponds to the crystalline-to-amorphous ratio, was calculated according to the Segal empirical method [36] by following the Equation (1):

(1) $$CrI\text{​}(\%)=\frac{\left(I_{002}-I_{am}\right)}{I_{002}}\times 100$$

where *I_002_* is the maximum intensity (in arbitrary units) of the diffraction from the (002) plane at 2θ = 22.6°; and *I_am_* is the intensity of the background scatter measured at 2θ = 19°.

#### 3.4.5. Mechanical Properties

For the analysis of mechanical properties, tensile tests based on UNE-EN ISO 13934-1 standard [37] were performed to determine the changes in the breaking force of the NW. The tests were performed in an MTS dynamometer (MTS Systems, Eden Prairie, MN, USA) with a load cell of 5 KN, at a displacement rate of 5 mm/min. The nonwovens were tested in the cross-direction.

## 4. Conclusions

In this study, different treatments were applied in flax-NW fabrics in order to evaluate their feasibility for composite reinforcement regardless of whether the matrix is hydrophobic or hydrophilic. On the one hand, the effectiveness of the wet/dry treatment in the achievement of greater stability against the water absorption of fibers has been observed. On the other hand, different strategies for the plasma treatments have shown very different results, thus indicating that the properties of the reinforcement can be adapted depending on the nature of the matrix. If hydrophilic reinforcement is required, Ar-plasma treatments have shown the most satisfactory results, even helping to enhance the mechanical properties of the NW. However, corona plasma treatments can also be considered, since a balance between cost-effectiveness and simplicity of the treatment can be achieved with fair properties. On the contrary, if hydrophobic reinforcement is required, the Et-plasma treatment of the flax-NW can be an interesting option, although a previous Ar-plasma or corona plasma treatment is recommended, since the latter seems to improve the effectiveness of the former.
